# A new weighted injury severity scoring system: better predictive power for adult trauma mortality

**DOI:** 10.1186/s40621-019-0217-8

**Published:** 2019-09-23

**Authors:** Junxin Shi, Jiabin Shen, Motao Zhu, Krista K. Wheeler, Bo Lu, Brian Kenney, Kathryn E. Nuss, Henry Xiang

**Affiliations:** 1 Center for Pediatric Trauma Research, The Abigail Wexner Research Institute at Nationwide Children’s Hospital, 700 Children’s Drive, Columbus, OH 43205 USA; 2 Center for Injury Research and Policy, The Abigail Wexner Research Institute at Nationwide Children’s Hospital, Columbus, OH USA; 3 The Ohio State University College of Medicine, Columbus, OH USA; 4 Department of Emergency Medicine, Nationwide Children’s Hospital, Columbus, OH USA; 5 Division of Biostatistics, The Ohio State University College of Public Health, Columbus, OH USA; 6 Department of Pediatric Surgery, The Ohio State University College of Medicine, Columbus, OH USA

**Keywords:** Injury severity score, Weighting, Adult, Trauma, Mortality

## Abstract

**Background:**

An accurate injury severity measurement is essential in the evaluation of trauma care and in outcome research. The traditional Injury Severity Score (ISS) does not consider the differential risks of the Abbreviated Injury Scale (AIS) from different body regions, and the three AIS involved in the calculation of ISS are given equal weights. The objective of this study was to develop a weighted injury severity scoring (wISS) system for adult trauma patients with better predictive power than the traditional Injury Severity Score (ISS).

**Methods:**

The 2007–2014 National Trauma Data Bank (NTDB) Research Datasets were used. We identified adult trauma patients from the NTDB and then randomly split it into a study sample and a test sample. Based on the association between mortality and the Abbreviated Injury Scale (AIS) from each of the six ISS body regions in the study sample, we evaluated 12 different sets of weights for the component AIS scores used in the calculation of ISS and selected one best set of weights. Discrimination (areas under the receiver operating characteristic curve, sensitivity, specificity, positive predictive value, negative predictive value, concordance) and calibration were compared between the wISS and ISS.

**Results:**

The areas under the receiver operating characteristic curves from the wISS and ISS are all 0.83, and 0.76 vs. 0.73 for patients with ISS = 16–74 and 0.68 vs. 0.53 for patients with ISS = 25–74. The wISS showed higher specificity, positive predictive value, negative predictive value, and concordance when they were compared at similar levels of sensitivity. The wISS had better calibration than the ISS.

**Conclusions:**

By weighting the AIS from different body regions, the wISS had significantly better predictive power for mortality than the ISS, especially in critically injured adults.

**Electronic supplementary material:**

The online version of this article (10.1186/s40621-019-0217-8) contains supplementary material, which is available to authorized users.

## Key points

### Question

Will a weighted Injury Severity Score (wISS) better predict mortality in adult trauma patients when compared with the traditional Injury Severity Score (ISS)?

### Findings

By weighting the Abbreviated Injury Scores (AIS) from different body regions, the wISS showed better predictive ability than the ISS, especially in critically injured adults (ISS in the range of 25–74).

### Meaning

The weighted ISS is a better alternative to ISS to predict mortality in adult trauma patients.

## Background

An accurate injury severity measurement is essential for evaluating trauma care and outcome research. Over the past four decades, the Injury Severity Score (ISS), developed by Susan Baker and colleagues in 1974 (Baker et al. 1974), has been the most commonly used injury severity measurement (Tohira et al. 2012). ISS is based on the severity score of the Abbreviated Injury Scale (AIS), which is an anatomically based consensus-derived severity scoring system that classifies each injury by body region and relative severity. A numerical scale of injury severity ranging from 1 (minor) to 6 (maximal severity) is assigned for each injury from six body regions ([1] Head/neck, [2] Face, [3] Chest, [4] Abdomen or pelvic contents, [5] Extremities or pelvic girdle, and [6] External) (Committee on Medical Aspects of Automotive Safety (CMAAS) 1971). An ISS (ranging from 1 to 75) is computed by summation of the squares of the three highest AIS scores from the most severely injured body regions. Any AIS score of 6 is assigned an ISS of 75, which suggests an un-survivable injury.

Although ISS is the most commonly used severity measurement, it has a critical limitation: it is weighted equally across all six body regions without consideration of the differing mortality risks from injuries in different body regions, leading to an inconsistent correlation between an ISS and mortality (Copes et al. 1988, Osler et al. 1996, Aharonson-Daniel et al. 2006). In our previous study, we developed a weighted Injury Severity Scoring system for pediatric blunt trauma as a proof of concept (Shi et al. 2018). The weighted ISS showed much better ability in predicting mortality in critically injured children (ISS ≥ 25). Because the first weighted ISS was developed in pediatric trauma patients, it is not clear if in adults, different weighting should be applied and whether weighting would then produce meaningful improvements in mortality prediction.

Since the inception of the ISS scoring system, a number of new injury severity scoring tools have been developed, including the New ISS (NISS) (Osler et al. 1997), the Trauma and Injury Severity Score (TRISS) (Champion et al. 1983), and the International Classification of Diseases (ICD)-based Injury Severity Score (ICISS) (Osler et al., 1996). However, these have not replaced the commonly used ISS. In the conclusion of a recent review paper, the authors report that the evidence is not consistent to support which score is superior (Tohira et al., 2012). The traditional ISS is still the only comprehensive injury severity measurement used in important national databases, such as the National Trauma Databank (NTDB) (American College of Surgeons 2007–2014) and the Nationwide Emergency Department Sample (NEDS) (The Healthcare Cost and Utilization Project (HCUP) 2006–2014).

In the pediatric paper, we used the NTDB for score development and the NEDS data to test the score. The majority of trauma patients included in NEDS data, especially the major trauma patients could be the same patients included in the NTDB data. So the sample for developing of the pediatric wISS and the sample for testing of the wISS most likely included many of the same patients. In this current adult paper, we have resolved this issue.

This study used a two-step procedure to accomplish its objective to develop and validate a new weighted ISS scoring system for adult trauma. We randomly split trauma patients identified from NTDB into a study sample and a test sample. We used the study sample to develop the best set of AIS weights for a weighted ISS. We then applied this set of weights to the test sample data to calculate the weighted ISS and compare the weighted ISS and the traditional ISS in terms of predicting mortality.

## Methods

### Data source

The 2007–2014 National Trauma Data Bank (NTDB) Research Datasets were used. The NTDB contains standardized trauma registry data from more than 900 trauma centers in the United States each year, and is the largest aggregation of U.S. trauma registry data (American College of Surgeons 2007–2014).

### Study population

Our study population was adult trauma patients (age ≥ 18 years and ISS 1–74). The following patients were excluded: patients who were transferred to another hospital; patients who arrived without signs of life; patients with ISS = 75; patients treated in hospitals without American College of Surgeons (ACS) Level I or Level II trauma center verification; patients without AIS scores submitted by the trauma centers. Those transferred out were excluded because their survival outcome was not known. Patients with an ISS = 75 were excluded, since their probability of death was close to certainty by definition, and it was impossible to separate patients with survivable and un-survivable injuries. We excluded patients not treated in ACS Level I/II centers, because those patients have higher mortality rates compared with Level I/II centers (MacKenzie et al. 2006), and we wanted the mortality rate of the sample to be a reflection of best practices. Another reason is that at Level I/II centers the AIS codes are likely coded by human instead of computer (American College of Surgeons 2014). AIS scores assigned by human coders have been found to be more accurate than computer software especially for patients with a higher risk of mortality (Shi et al., 2018). Patients with missing age, transfer status, sign of life at arrival, discharge disposition were also excluded. After applying our exclusion criteria, we randomly split the data into two halves, with one half as the score development sample and the other half as the test sample.

### Data analysis

Death was the main outcome and it included deaths occurring in the emergency department (ED) or during the hospital stay. All the analyses were done using SAS Enterprise Guide, Version 7.11 HF3 (SAS Institute Inc., Cary, NC, USA). There were two major steps in the data analysis:

### Step one: developing the weighted ISS

The weights for the AIS from the six ISS body regions should reflect the strength of the relationship between the AIS and mortality. We explored 12 sets of weights which can be divided into four weighting groups: Group A was based on the highest mortality of all AIS severity scores from each body region; Group B was based on overall mortality of injuries from each body region; Group C was based on the AUC (the area under the receiver operating characteristic curve); Group D was based on the concordance. Concordance is defined as the proportion of pairs where the observation with the event (death in this study) has a higher predicted probability (higher severity score) than the observation of non-event (survival) among all possible combinations of one event and one non-event. We further used two data transformations (the logarithm and square root). The choice of these two transformations is arbitrary. Although the two data transformation methods are commonly used in statistics, we do not know a priori whether these will generate optimal weights. The details are described as follows:

1. We identified the six maximum AIS severity scores (maxAIS) corresponding to the six ISS body regions from AIS scores included in the database (file: RDS_AIS98PCODE).
2. For each body region, we selected those patients with the maxAIS from that body region as the “principal injury,” and those patients with higher or equal maxAIS from other body regions were excluded temporarily in this score development step. For example, patients with a maximum head AIS of 3 and an abdominal AIS score of 3 or higher would be excluded when evaluating the relationship between head AIS and mortality in the development step. We calculated mortality rates correlated with each specific maxAIS value for each ISS body region and overall mortality rates for each ISS body region. For example, head injuries with maxAIS equal to 1, 2, 3, 4, 5, the mortality rates were 0.23, 0.76, 2.34, 5.45, and 40.81%, respectively in the study sample. The highest mortality rate was 40.81%, but the overall mortality rate for these patients was 7.84%. We fitted six logistic models with death as the outcome, and each of the six maxAIS as predictors to get the c statistic and the concordance (Since logistic regression is a monotonic transformation of the severity score, the c statistic from the logistic regression model is equivalent to the AUC calculated from the raw score). Compared to concordance, the c statistic takes into account those pairs where severity scores are equal for the pair of event and non-event (in other words, it includes adjustment by the ties).
3. We generated 12 sets of weights. Specifically,

A1: Highest mortality; A2: LOG (Highest mortality); A3: SQRT (Highest mortality);

B1: Overall mortality; B2: LOG (Overall mortality); B3: SQRT (Overall mortality);

C1: 100 × (AUC-0.5); C2: LOG(100 × (AUC-0.5)); C3: SQRT (100 × (AUC-0.5));

D1: Concordance; D2: LOG (Concordance); and D3: SQRT (Concordance).

LOG was the natural logarithmic function, and SQRT was the square root function. For the C1, C2, and C3 weighting methods, we subtracted 0.5 from the AUC to get a “net” discrimination (AUC = 0.5 is considered without any discrimination and just by chance).

(4) We applied (multiplied) the weights to the six maxAIS scores. We then squared the three largest numbers and added them together to get a weighted ISS. We also calculated the traditional ISS without applying weights.
(5) Selected the best set of weights by comparing AUCs.

Discrimination in this study is the ability of the score to separate the patients who survived and those who did not. In a Receiver Operating Characteristic (ROC) curve, the true positive rate (sensitivity) is plotted as a function of the false positive rate (1-specificity) for different cut-off points (Zweig and Campbell, 1993). The area under ROC curve (AUC) equal to 1 represents perfect discrimination; an AUC equal to 0.5 indicates no discriminative power. We compared the AUCs of all 12 weighting methods against the traditional ISS among various ranges of ISS. Ultimately, we chose the weighting method that maximized the AUC in major trauma (ISS 16–74). In this study, A3 (square root of the highest mortality) was chosen as the final weighting method.

### Step two: validating the weighted ISS (wISS) in the test sample

#### Discrimination comparisons

The set of weights we chose in the score development sample was applied to calculate the wISS in the test sample. We then compared the AUCs of the wISS and ISS in the test sample. In SAS, the comparison of AUCs implements the nonparametric approach of DeLong, DeLong, and Clarke-Pearson (DeLong et al. 1988). We also calculated sensitivity, specificity, positive predictive value (PPV), negative predictive value (NPV), and concordance with each individual score as the cutoff value in the range of ISS 1–74, major trauma (ISS 16–74), and in critically injured patients (ISS 25–74).

#### Calibration comparisons

Calibration is the ability of predictors to correctly predict an outcome over the entire range of risk. Calibration can be assessed graphically by plotting the actual outcome against the predictors. In our study, the ISS had 43 individual values while the wISS had over 1000 individual values. The smoothness of the curved line of the mortality rates in correspondence with the severity scores depends on the number of patients in each score group. If sample sizes for the groups were small, the line representing the mortality rates would oscillate wildly. If the number of groups was made smaller (i.e., the number of patients in each group will then be larger), the mortality rates tend to be stabilized. We generated scatter plots of mortality probabilities against wISS for different score groupings (i.e., on the original scale, with unit = 100, unit = 200, and by 5 groups: 0–400, 400–600, 600–1000, 1000–1400, 1400+). For the purpose of comparison, we generated similar graphs for the ISS, on the original scale and by 5 groups (1–3, 4–8, 9–15, 16–24, 25–74).

With the assumption that logistic regression accurately describes the relationship between mortality and the severity score, we examined the calibration indirectly by comparing the Hosmer and Lemeshow Goodness_of Fit Chi-square, with a smaller value indicating better fit.

## Results

A total of 4,777,423 adult trauma patients were identified in the 2007–2014 NTDB. We excluded in the following order, patients without AIS (27%), not treated in ACS Level I or Level II trauma centers (45.4%), transferred out (2.6%), arrival with no sign of life (0.6%), or ISS = 75 (0.2%). The final sample was 1,830,940. Females were 35%, and the average age was 49 years. Major trauma (ISS 15–74) patients were 24% of the sample, and 9% were critically injured (ISS 25–74). The mortality rate was 3.9% for the overall group, 4.5% for major trauma, and 26.2% for the critically injured patients. (Additional file 1: Table S1, Tables 1 and 3). In the study sample, the weighting method A3 (square root of the highest mortality) provided the highest discriminative power in the ISS range of 16–74, so we chose the weights from A3 as the final weights. The final weights were as follows: head/neck = 6.39; face = 4.18; chest = 4.80; abdomen/pelvic contents = 4.76; extremities = 5.65; external = 7.93 (Table 2). For the purpose of comparison, we have also listed the weights we generated in our pediatric blunt injury study in Table 2 (Shi et al., 2018). The other 11 sets of weights are shown in Additional file 1: Table S2, and the related AUCs from the study sample are shown in Additional file 1: Table S3.

**Table 1:** Demographic and injury characteristics of the sample

		Sample A: study sample		Sample B: test sample	
		n	%	n	%
Total		915,071		915,869	
Age	18-45 years	412,027	45.0	412,218	45.0
	45-64 years	259,642	28.4	260,21	28.4
	65+ years	243,402	26.6	243,440	26.6
Gender	Male	589,283	64.5	590,605	64.6
	Female	324,538	35.5	323,992	35.4
ISS	1-3	132,616	14.5	132,654	14.5
	4-8	274,155	30.0	273,288	29.8
	9-15	291,557	31.9	291,989	31.9
	16-24	136,481	14.9	137,083	15.0
	25-74	80,262	8.8	80,855	8.8
ACS Verification Level	I	569,979	62.3	570,894	62.3
	II	345,092	37.7	344,975	37.7
Mechanism	Blunt	771,243	84.3	770,884	84.2
	Penetrating	89,507	9.8	90,474	9.9
	Burn	14,940	1.6	14,881	1.6
	Other/unspecified	39,381	4.3	39,630	4.3
Isolated/Poly trauma	Isolated trauma	263,324	28.8	262,969	28.7
	Polytrauma	651,747	71.2	652,900	71.3

**Table 2:** Comparison of weights chosen for a pediatric (blunt trauma only) sample^a^ and the current adult sample (blunt, penetrating, and burns)

	Pediatric (Blunt trauma)^a^	Adult
Max AIS of head/neck	1.87	6.39
Max AIS of face	0.13	4.18
Max AIS of chest	1.52	4.80
Max AIS of abdomen/pelvic contents	0.98	4.76
Max AIS of extremities	0.15	5.65
Max AIS of external	0.33	7.93

^a^Source: Shi J, Shen J, Caupp S, Wang A, Nuss KE, Kenney B, Wheeler KK, Lu B, Xiang H. A new weighted injury severity scoring system: Better predictive power for pediatric trauma mortality. J Trauma Acute Care Surg. 2018;85(2):334-40

### Discrimination comparisons

All the subsequently described analyses are based on the test sample data. In patients with ISS 1–74, the AUCs from the weighted ISS and the traditional ISS are all 0.83. But in the ISS range of 16–74 (major trauma), the AUC from the weighted ISS is 0.76 while the AUC from the traditional ISS being 0.73; these two numbers are 0.68 vs. 0.53 in the range of ISS 25–74. The improvement in discrimination increased in the more severely injured patients. To study the potential generalization of the weighted ISS, we compared the AUCs in subgroups: Injury type (blunt trauma, penetrating trauma, burns), Isolated/poly (isolated trauma, polytrauma), and age groups (18–44, 45–64, 65+). In all subgroup analyses, similar patterns were seen, although the differences were smallest in burn patients and greatest for patients with penetrating trauma (Table 3).

**Table 3:** Area Under the ROC Curve for ISS and wISS in the test sample (NTDB 2007-2014)

						ISS		wISS		Increase in AUC
		ISS groups	# patients	# death	% death	AUC	95% CI	AUC	95% CI	
All Trauma	Total	1-74 (all severity)	915,869	36,187	4.0	0.83	(0.83-0.83)	0.83	(0.83-0.83)	0.00
		1-15	697,931	8,776	1.3	0.60	(0.60-0.61)	0.60	(0.60-0.61)	0.00
		16-74 (major)	217,938	27,411	12.6	0.73	(0.73-0.74)	0.76	(0.76-0.77)	**0.03**
		16-24	137,083	6,181	4.5	0.52	(0.52-0.53)	0.51	(0.50-0.51)	-0.02
		25-74 (critical)	80,855	21,230	26.3	0.53	(0.52-0.53)	0.68	(0.67-0.68)	**0.15**
Injury Type	Blunt	1-74 (all severity)	770,884	28,272	3.7	0.82	(0.82-0.82)	0.82	(0.82-0.83)	0.00
		1-15	576,492	7,064	1.2	0.60	(0.60-0.61)	0.60	(0.59-0.60)	0.00
		16-74 (major)	194,392	21,208	10.9	0.74	(0.74-0.74)	0.77	(0.76-0.77)	**0.03**
		16-24	124,796	4,995	4.0	0.50	(0.49-0.50)	0.52	(0.51-0.53)	0.02
		25-74 (critical)	69,596	16,213	23.3	0.56	(0.55-0.56)	0.68	(0.68-0.69)	**0.13**
	Penatrating	1-74 (all severity)	90,474	5,910	6.5	0.88	(0.88-0.89)	0.89	(0.89-0.90)	0.01
		1-15	74,895	1,077	1.4	0.61	(0.59-0.63)	0.62	(0.60-0.64)	0.01
		16-74 (major)	15,579	4,833	31.0	0.69	(0.69-0.70)	0.77	(0.77-0.78)	**0.08**
		16-24	7,303	885	12.1	0.59	(0.57-0.61)	0.52	(0.50-0.54)	-0.07
		25-74 (critical)	8,276	3,948	47.7	0.49	(0.48-0.50)	0.73	(0.71-0.74)	**0.23**
	Burn	1-74 (all severity)	14,881	618	4.2	0.87	(0.85-0.89)	0.87	(0.85-0.89)	0.00
		1-15	13,763	244	1.8	0.73	(0.70-0.76)	0.73	(0.70-0.76)	0.00
		16-74 (major)	1,118	374	33.5	0.69	(0.66-0.72)	0.70	(0.67-0.73)	**0.02**
		16-24	554	89	16.1	0.52	(0.48-0.56)	0.51	(0.47-0.54)	-0.02
		25-74 (critical)	564	285	50.5	0.49	(0.46-0.52)	0.53	(0.49-0.56)	**0.04**
Isolated/poly	Isolated trauma	1-74 (all severity)	262,969	5,473	2.1	0.74	(0.73-0.75)	0.75	(0.74-0.75)	0.01
		1-15	242,937	3,158	1.3	0.60	(0.59-0.61)	0.62	(0.61-0.63)	0.01
		16-74 (major)	20,032	2,315	11.6	0.75	(0.74-0.76)	0.76	(0.75-0.77)	**0.01**
		16-24	14,452	663	4.6	0.50	(0.50-0.50)	0.51	(0.50-0.53)	0.01
		25-74 (critical)	5,580	1,652	29.6	0.50	(0.50-0.50)	0.62	(0.61-0.63)	**0.12**
	Polytrauma	1-74 (all severity)	652,900	30,714	4.7	0.84	(0.84-0.84)	0.84	(0.84-0.84)	0.00
		1-15	454,994	5,618	1.2	0.61	(0.60-0.62)	0.61	(0.60-0.61)	0.00
		16-74 (major)	197,906	25,096	12.7	0.74	(0.73-0.74)	0.76	(0.76-0.77)	**0.03**
		16-24	122,631	5,518	4.5	0.52	(0.51-0.53)	0.51	(0.50-0.51)	-0.02
		25-74 (critical)	75,275	19,578	26.0	0.54	(0.54-0.55)	0.68	(0.67-0.68)	**0.13**
Age, years	18-44	1-74 (all severity)	412,218	11,713	2.8	0.86	(0.86-0.86)	0.86	(0.86-0.86)	0.00
		1-15	321,398	2,133	0.7	0.56	(0.55-0.57)	0.56	(0.55-0.57)	0.00
		16-74 (major)	90,820	9,580	10.6	0.76	(0.75-0.76)	0.78	(0.77-0.78)	**0.02**
		16-24	53,119	1,452	2.7	0.54	(0.52-0.55)	0.54	(0.53-0.56)	0.01
		25-74 (critical)	37,701	8,128	21.6	0.57	(0.56-0.58)	0.69	(0.68-0.69)	**0.12**
	45-64	1-74 (all severity)	260,211	9,212	3.5	0.84	(0.84-0.85)	0.85	(0.84-0.85)	0.00
		1-15	195,857	1,885	1.0	0.58	(0.57-0.59)	0.58	(0.57-0.60)	0.00
		16-74 (major)	64,354	7,327	11.4	0.76	(0.76-0.77)	0.79	(0.79-0.80)	**0.03**
		16-24	41,484	1,460	3.5	0.51	(0.50-0.53)	0.51	(0.50-0.53)	0.00
		25-74 (critical)	22,870	5,867	25.7	0.56	(0.55-0.56)	0.70	(0.69-0.71)	**0.14**
	65+	1-74 (all severity)	243,440	15,262	6.3	0.79	(0.78-0.79)	0.79	(0.78-0.79)	0.00
		1-15	180,676	4,758	2.6	0.59	(0.58-0.60)	0.59	(0.58-0.60)	0.00
		16-74 (major)	62,764	10,504	16.7	0.73	(0.73-0.74)	0.73	(0.72-0.74)	**0.00**
		16-24	42,480	3,269	7.7	0.52	(0.51-0.53)	0.52	(0.51-0.54)	0.00
		25-74 (critical)	20,284	7,235	35.7	0.54	(0.53-0.54)	0.64	(0.63-0.65)	**0.10**

*AUC* Areas under the receiver operating characteristic, *ISS* Injury severity score, *CI* Confidence interval

**Fig. 1:**
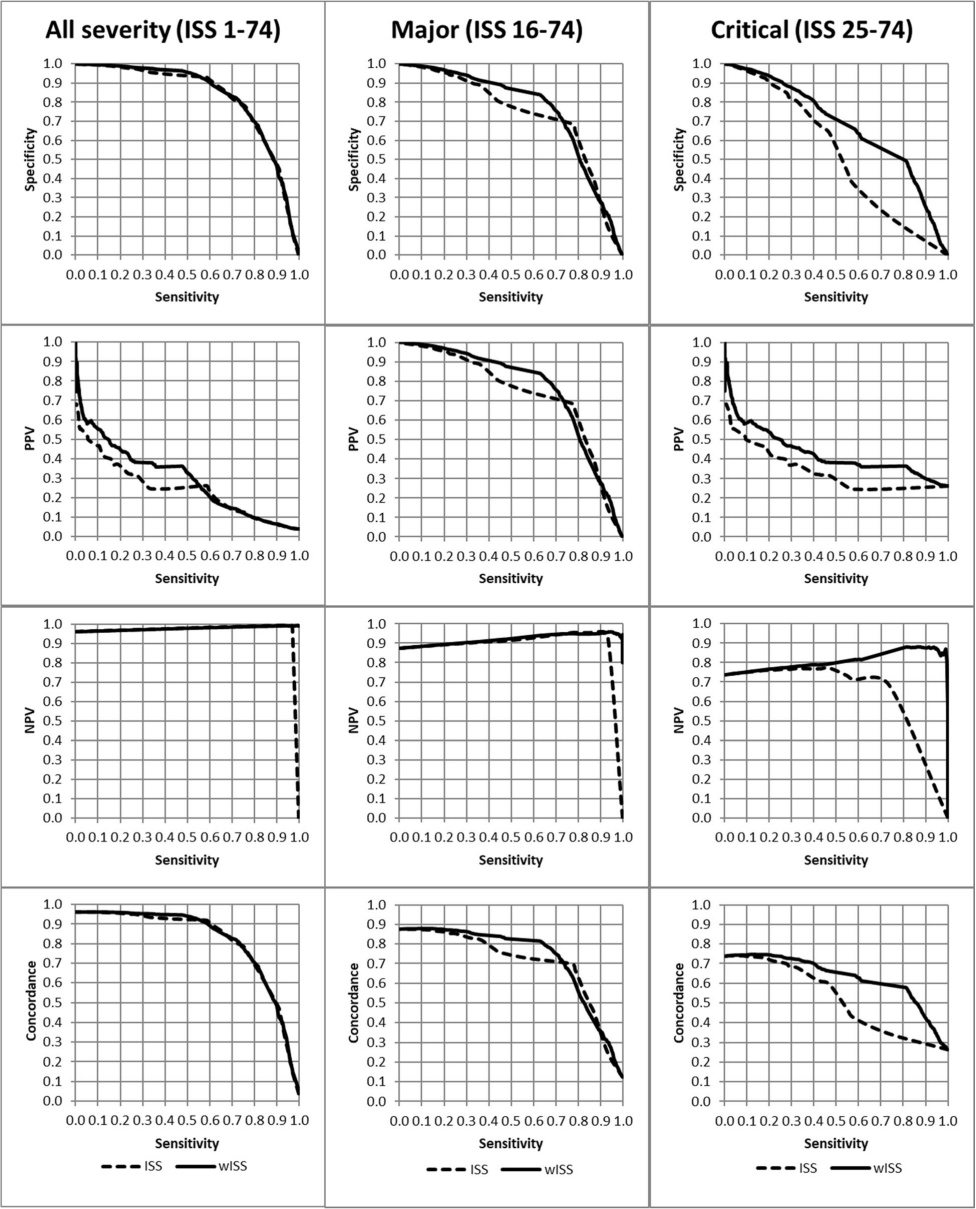
Comparing specificity, positive predictive value (PPV), negative predictive value (NPV), and concordance against sensitivity for the weighted injury severity score (wISS) and the injury severity score (ISS), in the range of all severity (ISS 11–74), major (ISS 16–74), and critical (ISS = 25–74), test sample NTDB 2007–2014. The weighted ISS (solid line) showed better diagnostic attributes in the major trauma group, especially in critically injured patients comparing to the traditional ISS (dashed line)

Figure 1 shows that with similar sensitivity, the specificity, positive predictive value, negative predictive value, and concordance were all higher for the wISS when compared with ISS in the major trauma group (ISS 16–74) and in critically injured patients (ISS 25–74).

### Calibration comparisons

**Fig. 2:**
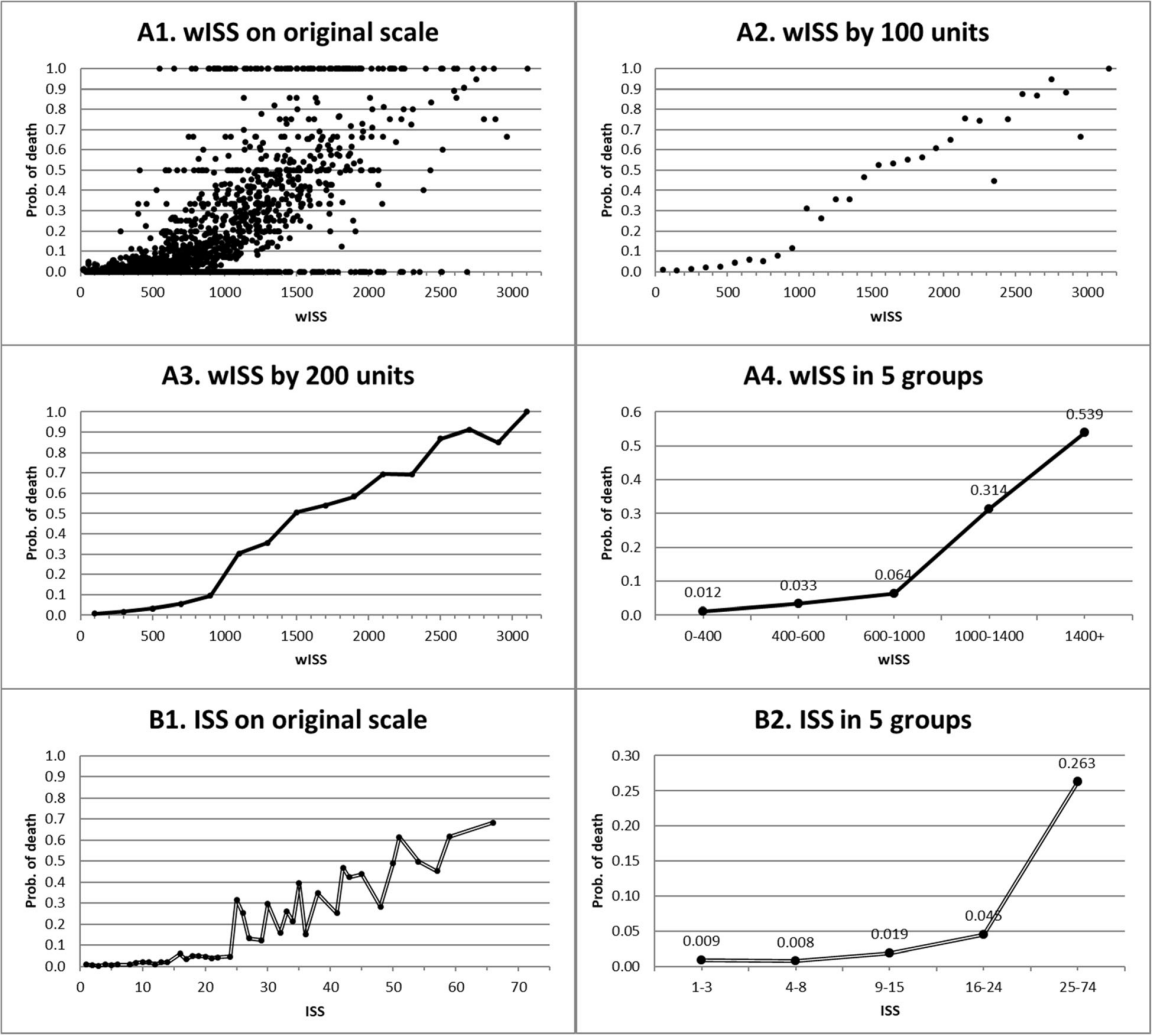
Observed mortalities by wISS and by ISS on different scales, test sample NTDB 2007–2014. **A1**-**A4** are for wISS, and **B1**-**B2** are for ISS. For ISS, the mortality rate in the range of ISS 1–3 is higher than in the range of ISS 4–8. Otherwise, the wISS and ISS showed mortality increasing along with score increasing

**Fig. 3:**
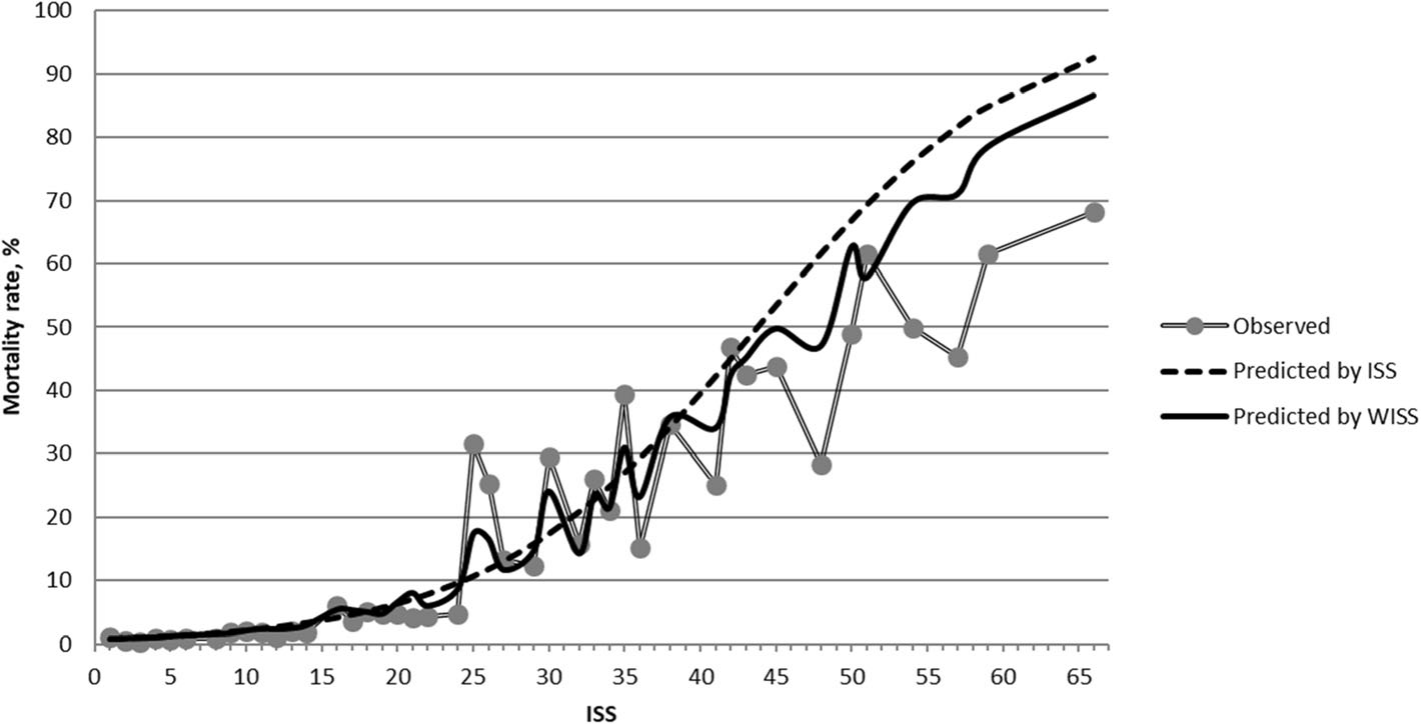
Comparison of goodness-of-fit between ISS and wISS in test sample, NTDB 2007–2014. Observed mortality and predicted by ISS and wISS are all grouped by individual ISS values. Overall, the predicted by wISS are closer to the observed comparing to ISS, especially in the range of critical injuries (ISS 25–74). Numerically, the Hosmer and Lemeshow Goodness-of-fit Chi-Square is 3441 for ISS and 432 for wISS (the overall difference between predicted and observed, smaller is better)

To compare calibration (how well the severity scores were associated with mortality), we present the data in two ways: (1) by drawing scatter plots for mortality rates against the scores, and (2) by comparing the goodness-of-fit of the logistic models. Scatter plots of mortality against severity scores (Fig. 2) showed that the trends were clearer with a decrease in the number of score groups. For ISS, the mortality rate in the range of ISS 1–3 was higher than in the range of ISS 4–8 while the wISS showed a monotonic trend (mortality increased along with score increases). It was difficult to discern which one was better in predicting mortality just by checking these graphs visually. However, the Hosmer and Lemeshow statistic was much smaller for the wISS model than for ISS (432 vs. 3441). Figure 3 depicts observed mortality rates along with the predicted mortality rates from ISS and wISS. The predicted mortality rates from the wISS were much closer to the observed mortality rates, especially in critically injured patients (ISS 25–74). We observed a non-monotonic curve representing the predicted mortality rates from the wISS, this is because a higher ISS does not always correspond to a higher wISS. But the most important task was to evaluate which one was closer to observed data points; in this case, it was notably the wISS. The closeness was represented by the Hosmer and Lemeshow statistics. Assuming the logistic regression models were correct, we can state that the wISS has a stronger association with mortality than ISS in trauma patients across the entire range of injury severity, but especially in critically injured patients.

## Discussion

We developed a weighted Injury Severity Score (wISS) by applying data derived weights to AIS scores to calculate the ISS. In a large sample of adult trauma patients, the wISS predicted mortality more accurately than the traditional ISS, especially in critically injured patients.

In the present study, we explored 12 weighting methods. The results confirmed our previous study developing a weighted ISS score for use in pediatric blunt trauma as well as other researchers’ results demonstrating that injuries from different body region should be given different weights while calculating ISS score (Copes et al., 1988, Cooper et al. 1994, Schneier et al. 2006, Brown et al. 2017, Shi et al. 2018). In pediatric patients, head injuries are weighted more than injuries from other body regions, but in our adult sample, the external injuries were given greater weighting than the head/neck injuries. Our current study results suggest that the weighting method to calculate ISS should be age specific, and at the very least children and adults should be treated differently. The weights developed in pediatric sample cannot be applied to adult sample, and vice versa.

The AUC is the most commonly used measurement of discrimination (Tohira et al., 2012). Most studies have included patients with all levels of injury severity, and the majority of the injured patients have minor injuries. Given this patient mix, the AUCs from many scoring systems tend to be high. In a study conducted by Meredith and colleagues, they compared nine scoring algorithms in predicting mortality, and their conclusion was that the differences in performance were relatively small (Meredith et al. 2002). In their study, the AUC for ISS was 0.867. The AUC can be intuitively understood in this way: if there is a pair of patients where one is randomly selected from the non-survival group and the other is randomly selected from the survival group, the AUC is equivalent to the probability of correctly identifying the non-surviving patient as the one with a higher severity score and the surviving patient as the one with a lower severity score (correctly classifying the two patients in the random pair). In this situation, if the majority of patients have minor injuries, it is not too difficult to correctly guess which one has a higher risk of death and which one has lower risk if you knew the scores of the pair, since the difference in terms of severity between these two patient pools is large. This is why many researchers previously have tried to develop alternative severity scores but ultimately failed to “improve” on the already very high AUC of the traditional ISS. Most studies did not focus on severe injured patients. The real challenge for a score is to discriminate among injured patients with high risk of mortality, and our weighted ISS performed much better than the traditional ISS in critically injured patients (ISS ≥ 25). In this ISS range, the ISS had very limited discrimination (AUC = 0.53) while the weighted ISS had an AUC 0.68. The improvement in discrimination and calibration strongly support our statement that the weighted ISS significantly improves upon the predictive capacity of mortality in critically injured patients when compared with the traditional ISS.

The results from our study have implications for risk adjustment in trauma outcome research and trauma program evaluation. The wISS could be used to risk-adjust in quality improvement efforts. The weighted ISS is a better alternative to ISS to predict mortality in adult trauma patients, especially when evaluating mortality risk of the most severe patients.

### Study limitations

Although this study successfully developed a new weighted ISS scoring system for adult trauma that performed better than the traditional ISS, our study has several limitations. First, we did not search all possible combinations of weights to optimize the prediction ability of the new score. The choice of the two methods of data transformations was arbitrary. Second, we did not compare the wISS with other scoring systems. Whether the wISS is better than other score is not yet known. Currently, ISS is the most commonly used severity measurement. As we indicated in above discussion, ISS is the only severity score used in two national databases (NTDB and NEDS). So, comparing with ISS is an important step and should be the first step when developing a new score. Finally, the calculation of the weighted ISS is more cumbersome than the traditional ISS, and most often will require the use of a computer and statistical programming. However, the computer algorithm could be provided upon request and that could then easily be incorporated into calculations.

## Conclusions

In summary, by weighting AIS scores from different body regions, the final weighted ISS had significantly better predictive power for mortality than the traditional ISS in critically injured adult trauma patients. This study demonstrates that consideration of the differing mortality risks from injuries in different body regions improves severity scoring. A wISS should be used in the evaluation of adult trauma care and in outcome research, since it is a significantly better predictor of mortality.

## Additional file

Additional file 1: **Table S1.** Mortality rates by ISS in adult trauma, NTDB 2007-2014. **Table S2.** Weights used by various weighting methods. **Table S3.** AUC comparisons between traditional ISS and 12 weighting methods in different ranges of ISS. (DOCX 2392 kb)

## Data Availability

The SAS programs generated the results are available to readers upon request.

## Abbreviations

AIS: Abbreviated Injury Scores
AUC: The Area under the Receiver Operating Characteristic curve
ICISS: International Classification of Diseases (ICD)-based Injury Severity Score
ISS: Injury Severity Score
NISS: New Injury Severity Score
NPV: Negative predictive value
NTDB: National Trauma Databank
PPV: Positive predictive value
ROC: Receiver Operating Characteristic
TRISS: Trauma and Injury Severity Score
wISS: weighted Injury Severity Score
