# Supplementary material for: A new weighted injury severity scoring system: better predictive power for adult trauma mortality
Source: Inj Epidemiol. 2019 Sep 23;6:40. doi: 10.1186/s40621-019-0217-8 (PMC6755696; doi:10.1186/s40621-019-0217-8)
Supplement: Supplementary file 1 [file 40621_2019_217_MOESM1_ESM.docx]

eTable 1. Mortality rates by ISS in adult trauma, NTDB 2007-2014

		Sample A: study sample				Sample B: test sample			
**ISS group**	**ISS value**	**# patients**	**# deaths**	**Mortality rate,%**	**Mortality rate by ISS group,%**	**# patients**	**# deaths**	**Mortality rate,%**	**Mortality rate by ISS group,%**
**ISS 1-3**	**1**	102,598	1,080	1.05	0.91	102,889	1,036	1.01	0.90
	**2**	26,420	107	0.40		26,277	146	0.56	
	**3**	3,598	16	0.44		3,488	13	0.37	
**ISS 4-8**	**4**	147,926	1,145	0.77	0.77	147,547	1,153	0.78	0.76
	**5**	93,623	669	0.71		93,450	631	0.68	
	**6**	14,101	98	0.69		14,051	111	0.79	
	**8**	18,505	207	1.12		18,240	171	0.94	
**ISS 9-15**	**9**	158,434	3,004	1.90	1.89	158,606	2,937	1.85	1.89
	**10**	60,562	1,175	1.94		60,434	1,232	2.04	
	**11**	6,985	134	1.92		6,952	133	1.91	
	**12**	7,143	65	0.91		7,213	80	1.11	
	**13**	26,672	565	2.12		26,601	522	1.96	
	**14**	31,761	575	1.81		32,183	611	1.90	
**ISS 16-24**	**16**	30,763	1,839	5.98	4.50	30,669	1,840	6.00	4.51
	**17**	45,965	1,640	3.57		46,164	1,608	3.48	
	**18**	10,853	489	4.51		10,803	541	5.01	
	**19**	8,353	368	4.41		8,393	401	4.78	
	**20**	8,302	374	4.50		8,276	381	4.60	
	**21**	11,816	503	4.26		11,925	480	4.03	
	**22**	13,685	579	4.23		13,918	604	4.34	
	**24**	6,744	346	5.13		6,935	326	4.70	
**ISS 25-74**	**25**	17,582	5,492	31.24	26.18	17,732	5,596	31.56	26.26
	**26**	13,300	3,459	26.01		13,240	3,341	25.23	
	**27**	5,001	622	12.44		5,041	669	13.27	
	**29**	13,131	1,585	12.07		13,363	1,656	12.39	
	**30**	2,746	851	30.99		2,808	829	29.52	
	**32**	440	70	15.91		458	72	15.72	
	**33**	2,321	535	23.05		2,303	601	26.10	
	**34**	7,101	1,502	21.15		7,226	1,529	21.16	
	**35**	1,943	731	37.62		1,945	767	39.43	
	**36**	1,927	278	14.43		1,940	294	15.15	
	**38**	3,822	1,352	35.37		3,899	1,351	34.65	
	**41**	2,671	696	26.06		2,612	657	25.15	
	**42**	459	207	45.10		498	233	46.79	
	**43**	2,230	919	41.21		2,161	916	42.39	
	**45**	1,582	668	42.23		1,605	703	43.80	
	**48**	281	72	25.62		269	76	28.25	
	**50**	2,316	1,176	50.78		2,239	1,097	49.00	
	**51**	90	50	55.56		96	59	61.46	
	**54**	262	126	48.09		287	143	49.83	
	**57**	393	216	54.96		442	200	45.25	
	**59**	427	244	57.14		455	280	61.54	
	**66**	237	160	67.51		236	161	68.22	
**ISS 75**	**75**	1,907	1,439	75.46		1,916	1,484	77.45	
**Total**		916,978	37,428	4.08		917,785	37,671	4.10	
**Total(excluding ISS=75)**		915,071	35,989	3.93		915,869	36,187	3.95	

Note: (1) ISS did not correspond with mortality monotonously; (2) There was a jump in mortality at ISS=25, from less than 5% to over 20%; (3)Although 8.8% patients were in the group of ISS 25-74, they accounted for 58% of the deaths (ISS=75 were excluded).

Weights calculations in study sample (ISS 16:74)

		Death					
		No	Yes	Total	Mortality,%	Concordant, %	c statistic
Max AIS of head/neck						77.40	0.85
	1	5,997	14	6,011	0.23		
	2	70,227	536	70,763	0.76		
	3	57,080	1,365	58,445	2.34		
	4	77,492	4,467	81,959	5.45		
	5	19,094	13,163	32,257	40.81		
	Sub Total	229,890	19,545	249,435	7.84		
Max AIS of face						36.20	0.59
	1	5,482	55	5,537	0.99		
	2	19,461	94	19,555	0.48		
	3	2,986	16	3,002	0.53		
	4	33	7	40	17.50		
	Sub Total	27,962	172	28,134	0.61		
Max AIS of chest						57.10	0.72
	1	2,014	32	2,046	1.56		
	2	14,289	216	14,505	1.49		
	3	69,158	1,570	70,728	2.22		
	4	19,872	1,536	21,408	7.17		
	5	4,059	1,216	5,275	23.05		
	Sub Total	109,392	4,570	113,962	4.01		
Max AIS of abdomen/pelvic contents						73.00	0.81
	1	2,703	27	2,730	0.99		
	2	18,684	165	18,849	0.88		
	3	15,106	299	15,405	1.94		
	4	7,328	841	8,169	10.30		
	5	2,585	759	3,344	22.70		
	Sub Total	46,406	2,091	48,497	4.31		
Max AIS of extremities						44.90	0.66
	1	8,978	20	8,998	0.22		

eTable 3. AUC comparisons between traditional ISS and 12 weighting methods in different ranges of ISS groups, study sample NTDB 2007-2014

	ISS: 1-74		ISS: 1-15		ISS: 16-74		ISS: 16-24		ISS: 25-74	
	**AUC**	**95% CI**	**AUC**	**95% CI**	**AUC**	**95% CI**	**AUC**	**95% CI**	**AUC**	**95% CI**
**ISS**	0.83	(0.82 - 0.83)	0.60	(0.59 - 0.61)	0.73	(0.73 - 0.74)	0.52	(0.52 - 0.53)	0.53	(0.52 - 0.53)
**A1**	0.82	(0.81 - 0.82)	0.58	(0.57 - 0.59)	0.74	(0.74 - 0.75)	0.50	(0.49 - 0.51)	0.70	(0.69 - 0.70)
**A2**	0.83	(0.83 - 0.83)	0.61	(0.60 - 0.61)	0.76	(0.76 - 0.77)	0.50	(0.49 - 0.51)	0.65	(0.64 - 0.65)
**A3**	**0.83**	**(0.83 - 0.83)**	**0.60**	**(0.59 - 0.61)**	**0.76**	**(0.76 - 0.77)**	**0.50**	**(0.50 - 0.51)**	**0.68**	**(0.68 - 0.68)**
**B1**	0.80	(0.80 - 0.81)	0.57	(0.56 - 0.57)	0.71	(0.71 - 0.71)	0.50	(0.49 - 0.50)	0.67	(0.66 - 0.67)
**B2**	0.81	(0.81 - 0.82)	0.57	(0.56 - 0.58)	0.73	(0.72 - 0.73)	0.50	(0.50 - 0.51)	0.67	(0.66 - 0.67)
**B3**	0.82	(0.82 - 0.82)	0.59	(0.58 - 0.59)	0.74	(0.73 - 0.74)	0.50	(0.49 - 0.51)	0.67	(0.66 - 0.67)
**C1**	0.83	(0.83 - 0.83)	0.61	(0.60 - 0.61)	0.76	(0.76 - 0.77)	0.51	(0.50 - 0.51)	0.64	(0.63 - 0.64)
**C2**	0.83	(0.83 - 0.83)	0.61	(0.60 - 0.61)	0.74	(0.74 - 0.75)	0.51	(0.50 - 0.52)	0.56	(0.56 - 0.57)
**C3**	0.83	(0.83 - 0.83)	0.61	(0.60 - 0.61)	0.75	(0.75 - 0.76)	0.50	(0.49 - 0.51)	0.59	(0.59 - 0.60)
**D1**	0.83	(0.82 - 0.83)	0.59	(0.59 - 0.60)	0.75	(0.75 - 0.75)	0.51	(0.50 - 0.52)	0.66	(0.65 - 0.66)
**D2**	0.83	(0.83 - 0.83)	0.61	(0.60 - 0.61)	0.75	(0.75 - 0.75)	0.50	(0.49 - 0.51)	0.59	(0.59 - 0.59)
**D3**	0.83	(0.83 - 0.83)	0.61	(0.60 - 0.61)	0.76	(0.76 - 0.76)	0.51	(0.50 - 0.51)	0.63	(0.63 - 0.64)

AUC: areas under the receiver operating characteristic; ISS: injury severity score; CI: confidence interval; A1-D3: 12 weighting methods.

A3 using the square roots of the highest mortalities as weights was chosen as the "best" set of weights among the 12 sets of weights. They generated the highest AUC in the range of ISS 16-74 (major trauma).
